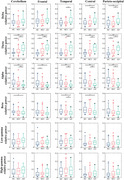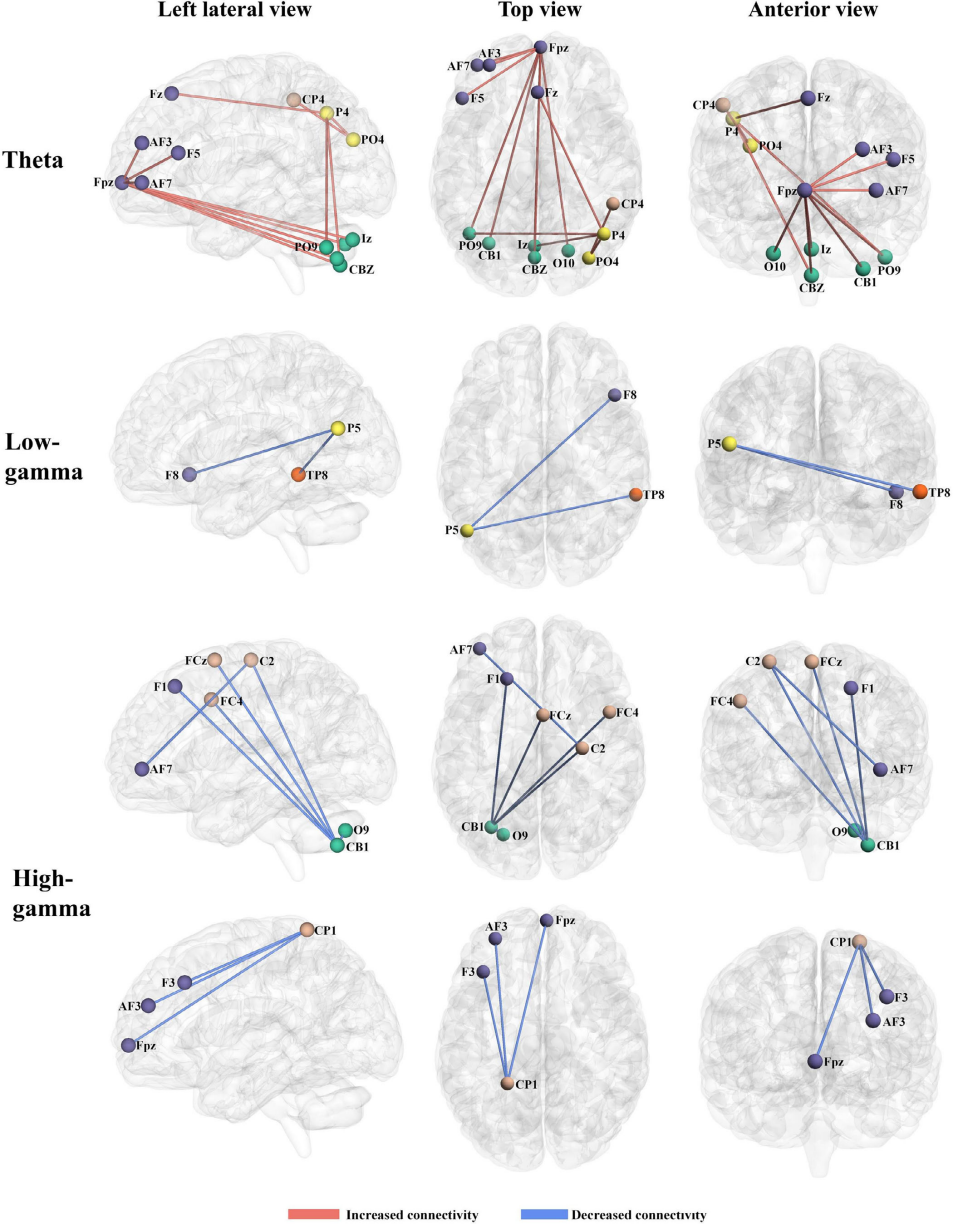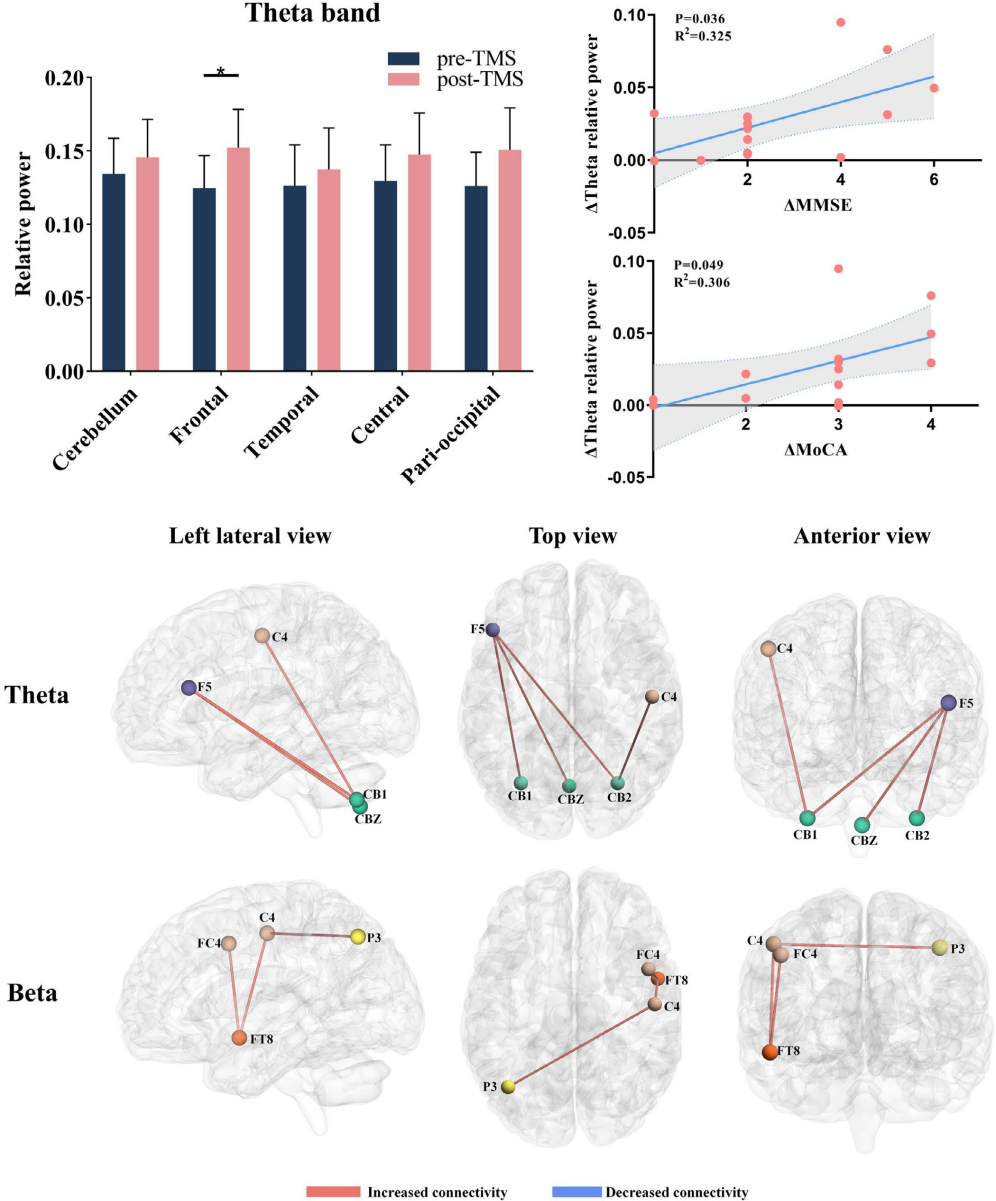# Electroencephalographic mechanisms of cognitive improvement induced by cerebellar stimulation in patients with mild cognitive impairment

**DOI:** 10.1002/alz70861_108711

**Published:** 2025-12-23

**Authors:** Chi Zhang, Minjie Tian, Jingping Shi

**Affiliations:** ^1^ Brain Hospital of Nanjing Medical University, Nanjing, Jiangsu China

## Abstract

**Background:**

The cerebellum has emerged as a key region involved in cognitive functions through cerebro‐cerebellar circuits. However, its electrophysiological features and the mechanisms underlying cerebellar repetitive transcranial magnetic stimulation (rTMS) in improving cognition remain unclear. This study investigated cerebellar electroencephalographic patterns in mild cognitive impairment (MCI) and explored the effects of bilateral cerebellar rTMS.

**Method:**

We recruited 31 healthy controls (HC), 41 MCI and 27 patients with Alzheimer's disease (AD) for this study. Fifteen MCI participants received 10‐day 5 Hz rTMS. Resting‐state electroencephalography(EEG) was recorded using 71 channels to monitor cerebrum and cerebellum electrical signals. We analyzed the spectral power and functional connectivity changes. Additionally, correlation analyses were conducted between spectral power and neuropsychological assessment scores.

**Result:**

MCI group showed significantly increased cerebellar theta band relative power compared to the HC group. Functional connectivity analysis indicated that enhanced functional connectivity was observed between the cerebellum and the left frontal lobe as well as the right parieto‐occipital region in the theta band. In the high‐gamma band, decreased functional connectivity was observed between the left cerebellum and the left frontal lobe, as well as the right central region. Cerebellar relative power in the theta band was negatively associated with MMSE and MoCA scores in the MCI group. After 5 Hz rTMS, a significant increase theta band relative power in frontal lobe was observed compared to the pre‐stimulation condition, accompanied by enhanced functional connectivity between the cerebellum and the left frontal lobe. Changes in theta band relative power were positively correlated with changes in MMSE and MoCA scores.

**Conclusion:**

The cerebellum relative power spectrum and functional connectivity in the theta band of resting‐state EEG holds promise as sensitive biomarkers for the early detection of MCI. Bilateral cerebellum 5 Hz rTMS enhanced theta band cerebellar relative power and functional connectivity between the cerebellum and the left frontal lobe, thereby augmenting the compensatory role of the cerebellum under cognitive impairment conditions and improving cognitive function in MCI.